# Practicability of clinical application of bladder cancer molecular classification and additional value of epithelial-to-mesenchymal transition: prognostic value of vimentin expression

**DOI:** 10.1186/s12967-020-02475-w

**Published:** 2020-08-05

**Authors:** João Lobo, Sara Monteiro-Reis, Catarina Guimarães-Teixeira, Paula Lopes, Isa Carneiro, Carmen Jerónimo, Rui Henrique

**Affiliations:** 1 Cancer Biology and Epigenetics Group IPO Porto Research Center (GEBC CI-IPOP), Portuguese Oncology Institute of Porto (IPO Porto) & Porto Comprehensive Cancer Center (P.CCC), R. Dr. António Bernardino de Almeida, 4200-072 Porto, Portugal; 2 Department of Pathology, Portuguese Oncology Institute of Porto (IPOP), R. Dr. António Bernardino de Almeida, 4200-072 Porto, Portugal; 3 Department of Pathology and Molecular Immunology, Institute of Biomedical Sciences Abel Salazar, University of Porto (ICBAS-UP), Rua Jorge Viterbo Ferreira 228, 4050-513 Porto, Portugal

**Keywords:** Bladder cancer, Molecular classification, Pathology, Luminal, Basal, Vimentin, EMT

## Abstract

**Background:**

Bladder cancer (BlCa) taxonomy has proved its impact in patient outcome and selection for targeted therapies, but such transcriptomic-based classification has not yet translated to routine practice. Moreover, epithelial-to-mesenchymal transition (EMT) has shown relevance in acquisition of more aggressive BlCa phenotype. We aimed to test the usefulness of the molecular classification, as defined by immunohistochemistry (a routinely performed and easy-to-implement technique), in a well-defined BlCa cohort of both non-muscle invasive (NMIBC) and muscle invasive (MIBC) disease. Also, we aimed to assess the additional prognostic value of the mesenchymal marker vimentin to the stratification strategy.

**Methods:**

A total of 186 samples were available. Immunohistochemistry/RT-qPCR for luminal markers GATA3/FOXA1, basal markers KRT5/KRT6A and vimentin were performed.

**Results:**

mRNA expression levels of the markers positively correlated with immunoexpression scores. We found substantial overlapping in immunoexpression of luminal and basal markers, evidencing tumor heterogeneity. In MIBC, basal tumors developed recurrence more frequently. NMIBC patients with higher vimentin immunoexpression endured poorer disease-free survival, and increased expression was observed from normal bladder-NMIBC-MIBC-metastases.

**Conclusions:**

The classification has the potential to be implemented in routine, but further adjustments in practical scoring should be defined; focusing on additional markers, including those related to EMT, may further refine BlCa molecular taxonomy.

## Background

Bladder cancer (BlCa) is one of the most incident cancers worldwide. It ranks ninth in prevalence, with a number of estimated new cases and cancer-related deaths of 549,393 and 199,922, respectively [1–3]. These figures are estimated to almost double by 2040 [1], representing an important toll on health services [4]. Most BlCa cases correspond to urothelial carcinoma, which is often divided into two major forms: 75–80% of all patients are diagnosed with non-muscle invasive BlCa (NMIBC), characterized by frequent recurrences and eventual progression to invasion; and the remaining 20–25% patients present with muscle-invasive BlCa (MIBC), which constitutes an aggressive, locally invading carcinoma, with propensity for metastization [5, 6]. On the therapeutic front, the clinical management of NMIBC and MIBC cases is very distinct, and it remained almost unchanged until the approval of immune checkpoint inhibitors in first-line or metastatic settings [7–9]. Nevertheless, a considerable percentage of BlCa patients do not benefit from current treatment options. Clinicians still have to deal with a high number of cases with recurrence and progression and, as a result, patients endure a long follow-up, making BlCa one of the costliest malignancies worldwide [4]. Hence, there is a need to improve risk stratification of these patients and to uncover biomarkers that may better select patients to the specific therapy that will give the higher benefit with less toxicity. In this line, an effort has been made to improve BlCa classification; various research teams have reported the importance of a molecular stratification of BlCa, and presented classifications based on different molecular traits, either for all urothelial carcinomas, or focusing on NMIBC and MIBC separately [10–20]. This molecular stratification is also useful for predicting responses to current treatment options, and provides insights for the development of new therapies [14, 21–24]. Although specific differences in classification emerge out of each research group analyses, they all share as an overlapping feature the existence of two major BlCa subtypes—basal/squamous and luminal—for MIBC cases [25]. Briefly, basal/squamous subtype is mainly composed of advanced stage tumors and metastatic disease, being enriched in inactivating mutations and deletions of *TP53* and *RB1*, whereas the luminal subtype is associated with papillary histopathological features, and enriched in fibroblast growth factor receptor 3 (*FGFR3*) mutations [26, 27]. An effort has been made to reach a single consensus classification and to generate a list of specific biomarkers (such as *FOXA1*, *GATA3*, *KRT5*/*6* and *KRT14*) that can be effectively translated from wide screening genomic and transcriptomics analyses into the clinic for any BlCa setting (both MIBC or NMIBC) [13, 26]. However, to date, this has not been achieved. On the other hand, the role of epithelial-to-mesenchymal transition (EMT) in BlCa prognosis has been widely discussed [28]. It has been shown to be highly related to an aggressive tumor biology, culminating in poor clinical outcome both in NMIBC and MIBC, namely poorer survival, increased recurrences, propensity to metastasize, and inferior response to treatment [29–33].

Herein, we aimed to characterize the expression of a set of markers for defining both luminal and basal/squamous subtypes in a well characterized patient cohort of BlCa, looking for clinicopathological correlates and testing their potential for clinical application, both within MIBC and NMIBC cases. Moreover, we explored the value of adding the expression of a classic EMT marker, vimentin (*VIM)*, to the risk stratification strategy. We have chosen *VIM* because among the EMT markers it is routinely performed in all Pathology departments and it has been consistently associated with BlCa prognosis, including in our previous in silico analysis [28].

## Methods

### Patients and samples

126 patients with primary BlCa (urothelial carcinoma) treated with transurethral resection (TUR) or radical cystectomy/cystoprostatectomy between 1991 and 2011 at the Portuguese Oncology Institute of Porto (IPO Porto) were retrospectively selected for the study. A set of 25 morphologically normal bladder mucosa tissue samples was obtained from BlCa-free individuals (prostate cancer patients submitted to radical prostatectomy with no bladder lesions) and served as controls. Additionally, a total of 35 metastases from BlCa were also included in the study. All specimens were formalin-fixed and paraffin-embedded for routine pathological examination by a dedicated uropathologist and used for immunohistochemistry studies. For some patients (see detailed numbers below) freshly collected tissue could be additionally obtained (a section matching the one embedded in paraffin). These were stored immediately at − 80 °C after surgical intervention and subsequently cut in a cryostat for confirmation of representativity. These freshly collected samples were specifically used for nucleic acid extraction (for mRNA expression analyses). Staging was performed using the American Joint Committee on Cancer (AJCC) 8th Edition manual [34]. Relevant clinical data was collected from clinical charts, by an investigator blinded to other study findings. A summary of the study cohort is presented in Table 1.

**Table 1:** Clinicopathological features of the study cohort

Clinicopathological features of the immunohistochemistry cohort	Primary bladder cancer
Individuals, n	126
Gender, n (%)	
Male	101 (80.2)
Female	25 (19.8)
Median age, years (range)	71 (61–77)
Grade, n (%)	
Papillary, low-grade	28/126 (22.2)
Papillary, high-grade	20/126 (15.9)
Invasive, high-grade	78/126 (61.9)
Pathological Stage, n (%)^a^	
pTa/pT1 (NMIBC)	51/123 (41.5)
pT2-4 (MIBC)	72/123 (58.5)

*NMIBC* non-muscle invasive bladder cancer, *MIBC* muscle invasive bladder cancer

^a^For 3 patients stage could not be ascertained as clinical data was missing/not available to the investigators

Patients and controls were enrolled after informed consent. This study was approved by the institutional review board (Comissão de Ética para a Saúde) of IPO Porto (CES103-14).

### Immunohistochemistry

In total, 186 samples were available for immunohistochemistry studies: the 126 primary BlCa specimens, plus the 25 normal bladder mucosae and 35 BlCa metastases. Immunohistochemistry methods are described in detail in Additional file 1: Table S1. Briefly, three micrometer-thick tissue sections from the formalin-fixed and paraffin-embedded samples were ordered, antigen retrieval was performed, and slides were incubated with the primary antibodies for FOXA1, GATA3, CK5/6 and VIM. Then, 3,3′-diaminobenzidine (Sigma-Aldrich™) was used as chromogen for visualization and slides were counterstained with hematoxylin. Appropriate tissue controls were used per run.

Immunoexpression patterns were evaluated by a dedicated uropathologist. Cases were classified using a semi-quantitative scale for both staining intensity (0—no staining; 1—low intensity, only barely discernible at 400 × magnification; 2—moderate intensity, well appreciated at 400× magnification but faint at 100× magnification; 3—high intensity, strong and well appreciated at 40× magnification) and percentage of positive cells (0— < 10%; 1—10–33%; 2—33–67%; 3— > 67%), in each case. Results were then combined in a single continuous score (Score S = staining intensity × percentage of positive cells) assigned to each tumor.

BlCa specimens were considered “basal-like” when at least focal positivity for CK5/6 was detected (independently of positivity for FOXA1 or GATA3), with the remaining samples (with complete absence of expression of CK5/6) being considered “luminal-like”, following the classification of Choi et al., centered on basal keratin expression for defining subtypes [22].

### Real-time quantitative PCR (RT-qPCR)

As mentioned, mRNA expression analyses were performed on fresh frozen tissues, available for 108 of the patients included in the study (all were run for *VIM* expression, and 83 for *FOXA1*, *GATA3*, *KRT5* and *KRT6A*, due to sample limitation issues). RNA was extracted from tissues using TRIzol^®^ (Invitrogen, Carlsbad, CA, USA), according to manufacturer’s instructions. RNA quantification and purity were assessed in NanoDrop™ Lite Spectophotometer (Cat. ND-LITE, Thermo Scientific™). cDNA synthesis was performed using the RevertAid™ RT Reverse Transcription Kit (Cat. K1691, Thermo Scientific™). The reaction was performed in MyCycler™ Thermal Cycler System (Cat. 1709703, Bio-Rad) using the following conditions: 5 min at 25 °C, 60 min at 42 °C and 5 min at 70 °C. *VIM* mRNA expression levels were evaluated using 4.5 µL of diluted cDNA, 5 µL of TaqMan^®^ Universal PCR Master Mix No AmpErase^®^ UNG (Applied Biosystems^®^) and 0.5 µL of TaqMan^®^ Gene Expression Assay, specific for *VIM* gene—assay ID Hs00185584. For normalization purposes, two TaqMan^®^ Gene Expression assays were used as internal controls: beta-glucoronidase—*GUSB*—assay ID Hs99999908, Applied biosystems^®^; and Hypoxanthine–guanine phosphoribosyltransferase—*HPRT1*—assay ID Hs01003267. RT-qPCR was run in 96-well plates, in an ABI 7500 Real Time PCR System (Thermo Fisher) in the following conditions: 2 min at 50 °C, followed by enzyme activation for 10 min at 95 °C, and 45 cycles which included a denaturation stage at 95 °C for 15 s and an extending stage at 60 °C for 60 s. Serial dilutions of cDNA obtained from Human Reference Total RNA (Cat. 750500, Agilent Technologies^®^) were used to compute standard curves for each plate. All experiments were run in triplicate and two negative controls were included in each plate. Relative expression of target genes tested in each sample was determined as: [Gene Expression Level = (Gene Mean Quantity/(*HPRT1* & *GUSB*) Mean Quantity) × 1000].

For *GATA3*, *FOXA1*, *KRT5* and *KRT6A* genes, transcript levels were also assessed using 2.5 µL of diluted cDNA, 0.25 µL of forward and reverse primers (Additional file 2: Table S2), 5 µL of Xpert Fast SYBER Mastermix Blue (GRiSP Research Solutions, Porto, Portugal) and 2 µL of bidistilled water. *GUSB* was used for normalization and plates were set as described above. The run followed the following conditions: 2 min at 95 °C, followed by 45 cycles of 5 s at 95 °C and 30 s at 60 °C, followed by the melt curve stage.

### Statistical analysis

Data was tabulated using Microsoft Excel 2016 and analyzed and plotted using GraphPad Prism 6 and IBM Statistical Package for Social Sciences (SPSS v24). Percentages were calculated based on the number of cases with available data. Individual data points are plotted, together with median and interquartile range. Mann–Whitney and Kruskal–Wallis tests were used for comparing expression levels among samples, as necessary. p-values were adjusted for multiple comparisons using Dunn’s test. Chi square and Fisher exact test were used as necessary for establishing associations between categorical variables. Spearman correlation test was used to correlate continuous variables. Disease-specific survival (DSS) and disease-free survival (DFS) curves were plotted using Kaplan–Meier statistics, and Cox regression models with respective hazard ratios (HR) were computed, including multivariable analysis. Statistical significance was set at p < 0.05.

## Results

### Clinical outcome of “luminal-like” and “basal-like” BlCa patients as determined by immunohistochemistry

There were no significant differences between the age distribution of patients with NMIBC and MIBC (p = 0.951). A total of 56/126 (44.4%) BlCa specimens showed “basal-like” features (following the Choi et al. stratification strategy, based on CK5/6 expression [22]). This occurred more frequently in MIBC (34/72, 47.2%) compared to NMIBC (20/51, 39.2%). However, 51/56 (91.1%) of the cases showing CK5/6 immunoexpression also exhibited immunoexpression of at least one of the markers GATA3/FOXA1, evidencing that most tumors show evidence of staining for both kinds of markers, in scattered cells. Four tumors showed no immunoexpression of either CK5/6, FOXA1 or GATA3 (three of those being MIBC) (Table 2). For the latter, we performed additional immunohistochemistry for neuroendocrine markers to look for the presence of the neuroendocrine-like molecular type of BlCa [10]. Indeed, one of the cases showed clear-cut strong immunoexpression of neuroendocrine markers synaptophysin, chromogranin and CD56 (Additional file 3: Fig. S1).

**Table 2:** Immunoexpression of luminal and basal markers in the bladder cancer cohort

	GATA3 and FOXA1 −	GATA3 and/or FOXA1 +
WHOLE COHORT		
CK5/6 −	4 (3.1%)	66 (52.4%)
CK5/6 +	5 (4.0%)	51 (40.5%)
NMIBC		
CK5/6 −	1 (2.0%)	30 (58.8%)
CK5/6 +	1 (2.0%)	19 (37.2%)
MIBC		
CK5/6 −	3 (4.2%)	35 (48.6%)
CK5/6 +	4 (5.5%)	30 (41.7%)

*MIBC* muscle invasive bladder cancer, *NMIBC* non-muscle invasive bladder cancer

For MIBC, there was no significant association between the luminal/basal-like subtype (as defined by immunohistochemistry, described above) and the event of metastization (p = 0.933). Within NMIBC, the “basal-like” cases disclosed disease recurrence in 8/20 cases (40.0%) and the “luminal-like” in a similar proportion of cases (13/31, 41.9%). However, considering MIBC, “basal-like” cancer developed recurrence in 11/34 cases (32.4%), whereas in “luminal-like” this occurred in a lower proportion of patients [only 5/38 cases (13.2%)].

**Fig. 1:**
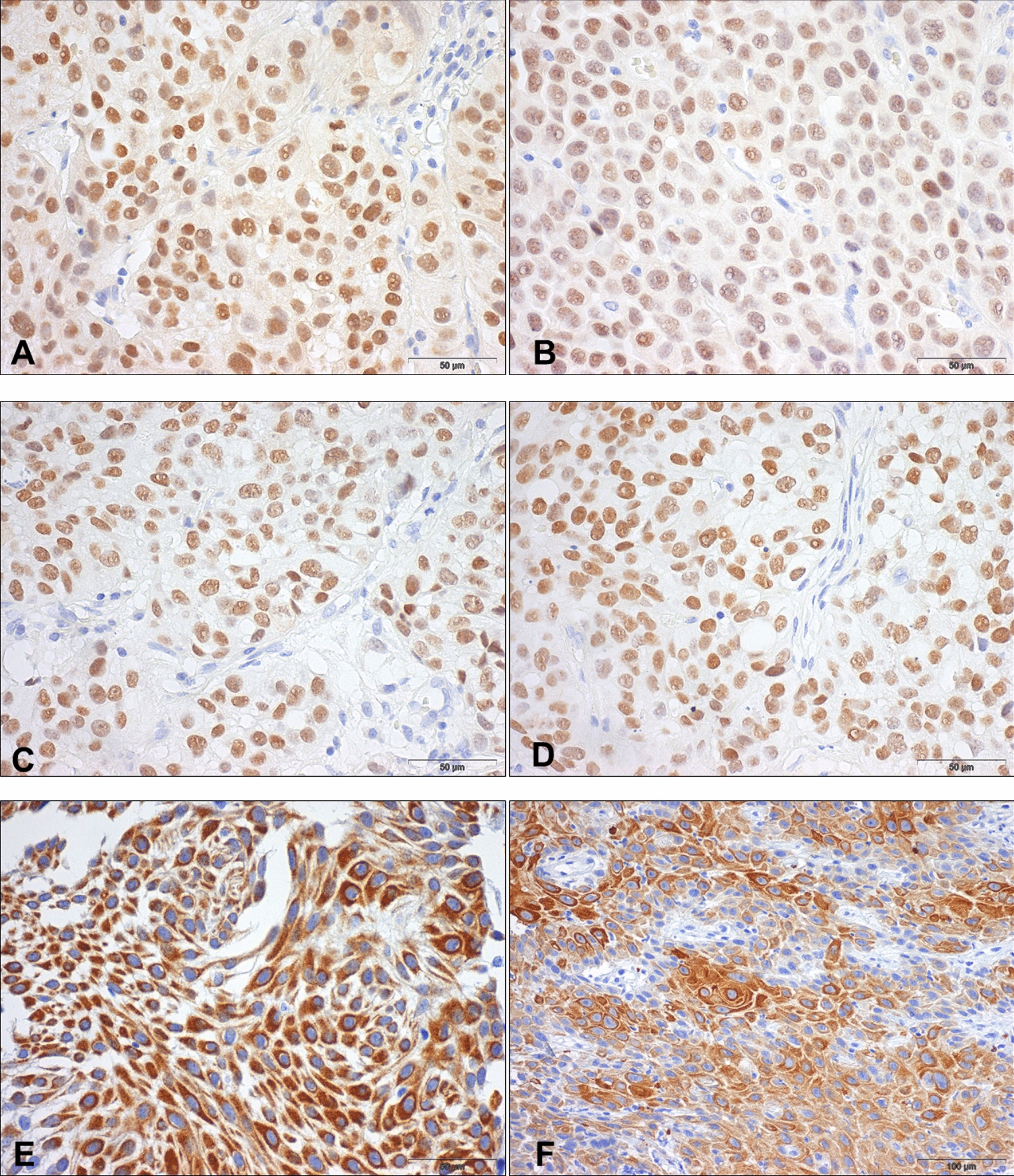
Immunoexpression of luminal and basal markers in the bladder cancer cohort. **a**, **b** FOXA1 strong and diffuse immunoexpression in two bladder cancer specimens, one NMIBC (**a**) and one MIBC (**b**); **c**, **d**: GATA3 strong and diffuse immunoexpression in two bladder cancer specimens, one NMIBC (**c**) and one MIBC (**d**); **e**, **f**: CK5/6 strong multifocal immunoexpression in two bladder cancer specimens, one NMIBC (**e**) and one MIBC (**f**)

Concerning survival analyses, the luminal/basal-like classification did not show significant impact on DSS or DFS, both for NMIBC or MIBC (NMIBC: p = 0.762 and p = 0.625; MIBC: p = 0.346, p = 0.185, respectively). Illustrative examples of immunoexpression patterns for the several markers are depicted in Fig. 1.

### Correlation between luminal/basal markers mRNA expression and protein expression

**Fig. 2:**
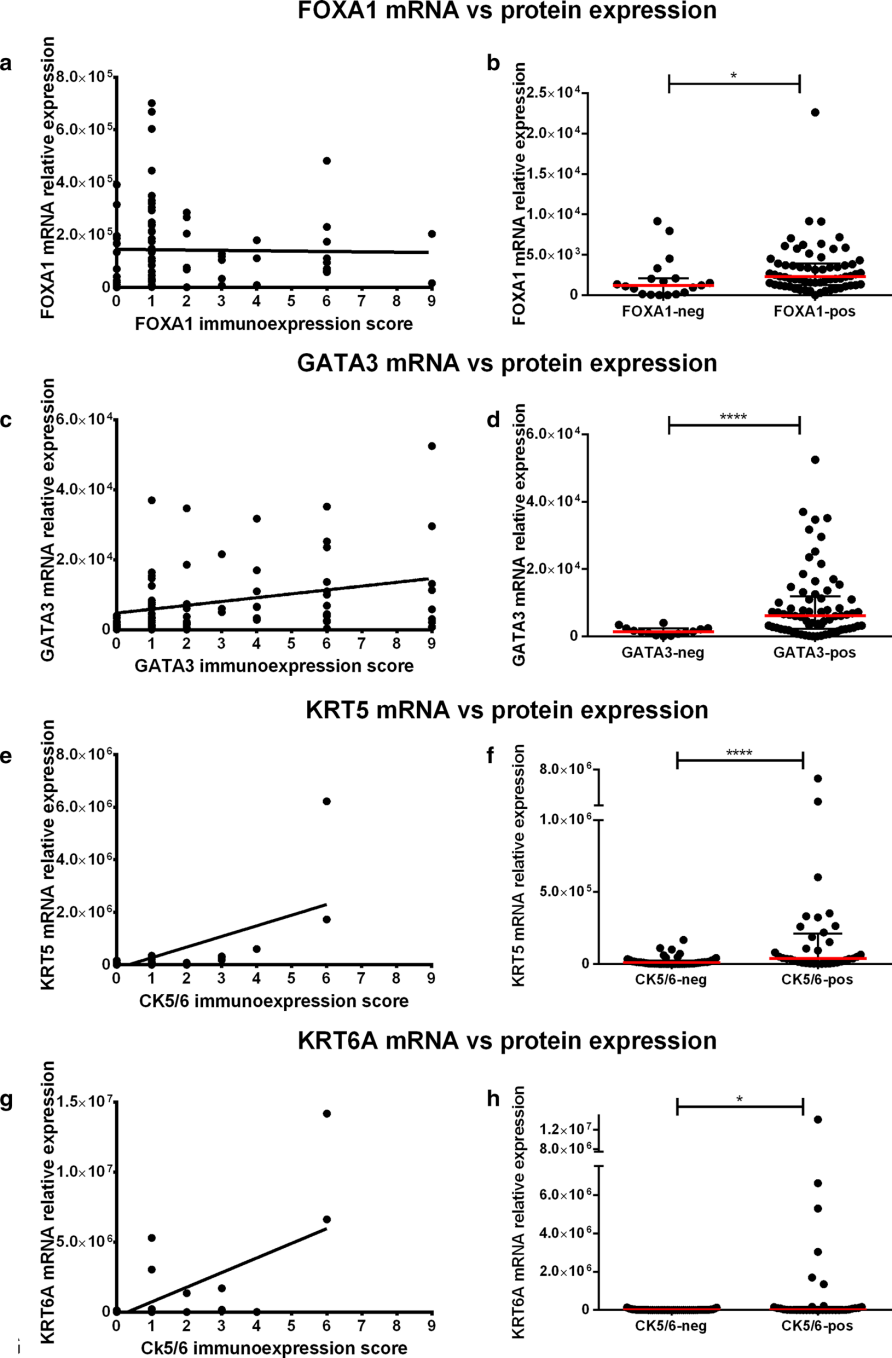
Correlation between mRNA and protein expression of the several luminal and basal markers in the bladder cancer cohort (both MIBC and NMIBC included). FOXA1 (**a** and **b**), GATA3 (**c** and **d**), KRT5 (**e** and **f**) and KRT6A (**g** and **h**) analyses. mRNA expression levels are plotted as relative expression levels, normalized to GUSB. Red dash and bars represent median and interquartile range. The immunoexpression score (intensity × percentage) is plotted in the xx-axis. The graphs include n = 83 matched samples (*p < 0.05; ****p < 0.0001)

We then checked for reproducibility between protein and transcript levels of the markers under study. Importantly, we found a significant, positive (albeit moderate), correlation between transcript levels of *GATA3* and its protein expression as assessed by immunoexpression score (r = 0.36, p = 0.010). However, the same was not found for *FOXA1* (r = 0.10, p = 0.3460). For basal markers *KRT5* and *KRT6A*, mRNA expression showed a significant positive, also moderate, correlation with the immunoexpression score (r = 0.49, p < 0.0001; and r = 0.68, p < 0.0001). Tumor samples with absent immunoexpression of GATA3, FOXA1 and CK5/6 showed significantly lower transcript levels of *GATA3*, *FOXA1* and *KRT5/KRT6A*, respectively (p < 0.001, p = 0.0130, p < 0.0001 and p = 0.0278) (Fig. 2).

### Additional value of VIM expression in predicting clinical outcome

**Fig. 3:**
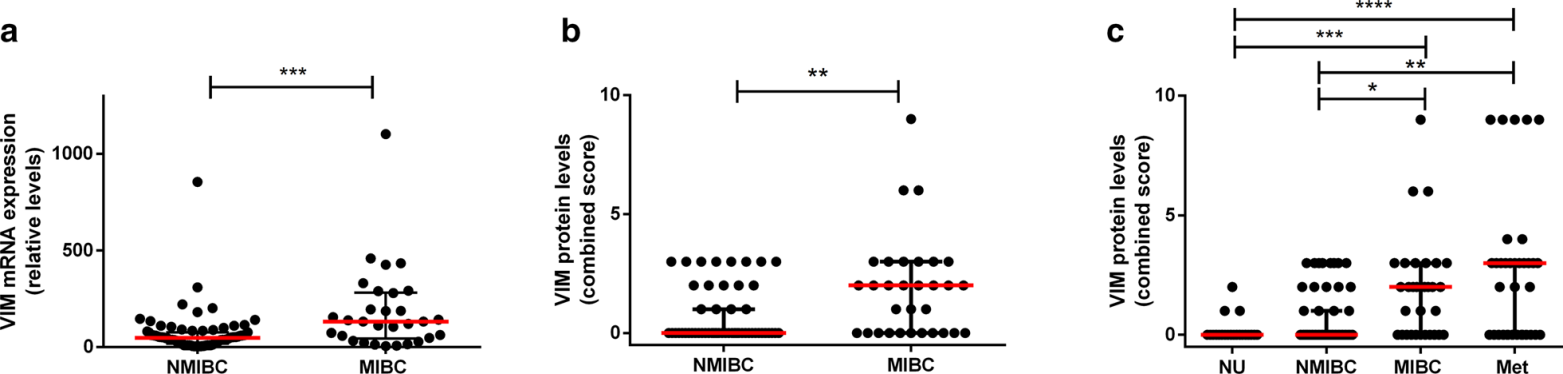
Vimentin transcript and protein levels within the bladder cancer cohort. **a** differential mRNA expression of vimentin between non-muscle (NMIBC) and muscle-invasive (MIBC) bladder cancer. mRNA expression levels are plotted as relative expression levels, normalized to GUSB and HPRT1; **b** differential protein (immuno)expression of vimentin between NMIBC and MIBC. The immunoexpression score (intensity × percentage) is plotted; **c** immunoexpression of vimentin among normal bladder, NMIBC, MIBC and bladder cancer metastases. The immunoexpression score (intensity × percentage) is plotted. Red dash and bars represent median and interquartile range. Correction for multiple comparisons was employed and adjusted p-values are represented (*p < 0.05; **p < 0.01; ***p < 0.001; ****p < 0.0001)

*VIM* transcript levels were significantly higher in MIBC compared to NMIBC (p = 0.0001, Fig. 3a). This was additionally validated at protein level by immunohistochemistry (p = 0.0013, Fig. 3b). Moreover, there was an overall progressive increase in immunoexpression scores for VIM, which were lower in normal urothelium and NMIBC, followed by MIBC, and attained the highest levels in BlCa metastases (p < 0.0001, Fig. 3c). Specifically, VIM immunoexpression scores were significantly higher in MIBC and metastases compared to normal urothelium and to NMIBC (after correction for multiple comparisons), however, differences between normal urothelium and NMIBC categories did not reach statistical significance (Fig. 3c).

**Fig. 4:**
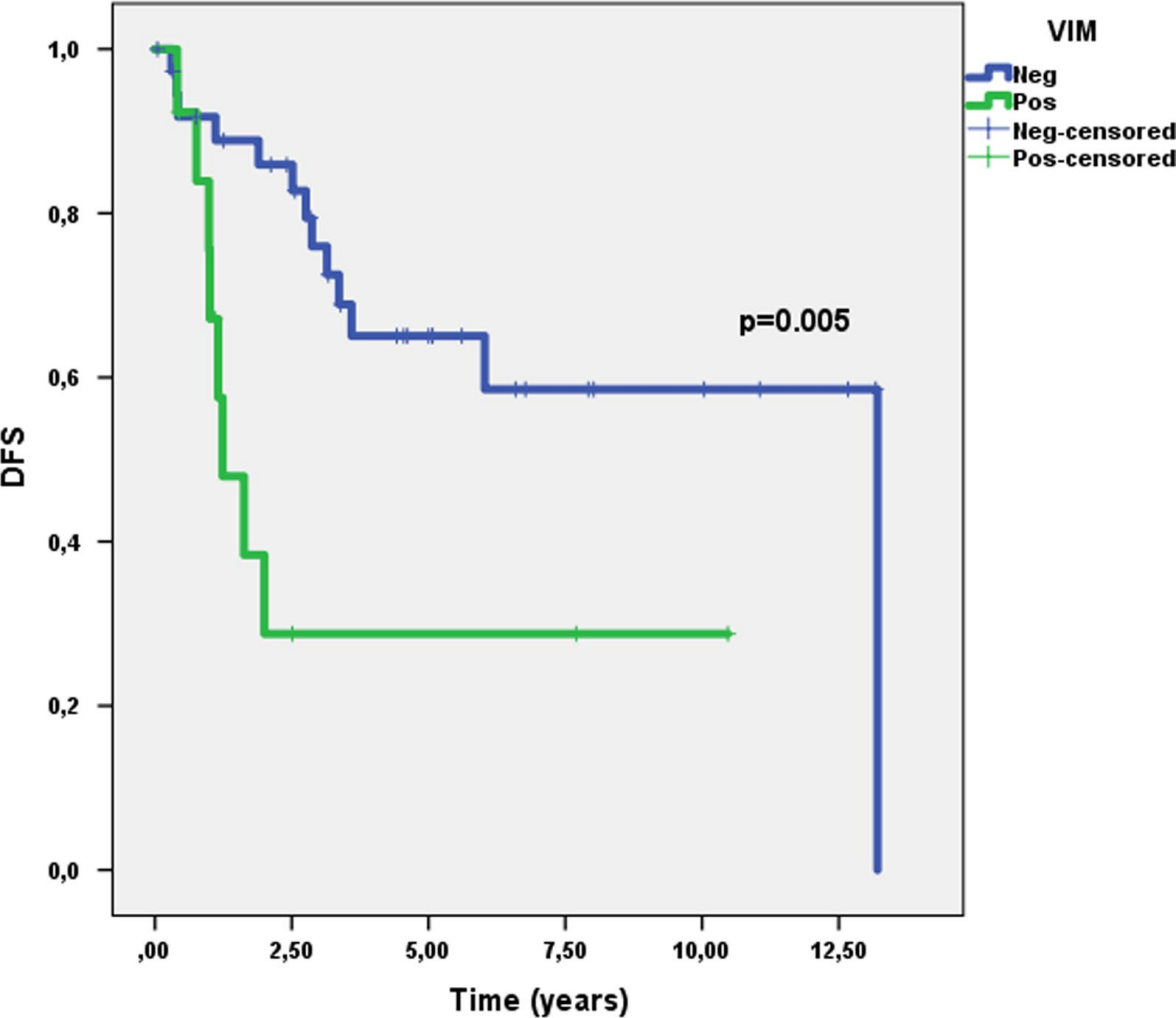
Disease-free survival in non-muscle invasive bladder cancer (NMIBC) patients according to vimentin protein expression

**Fig. 5:**
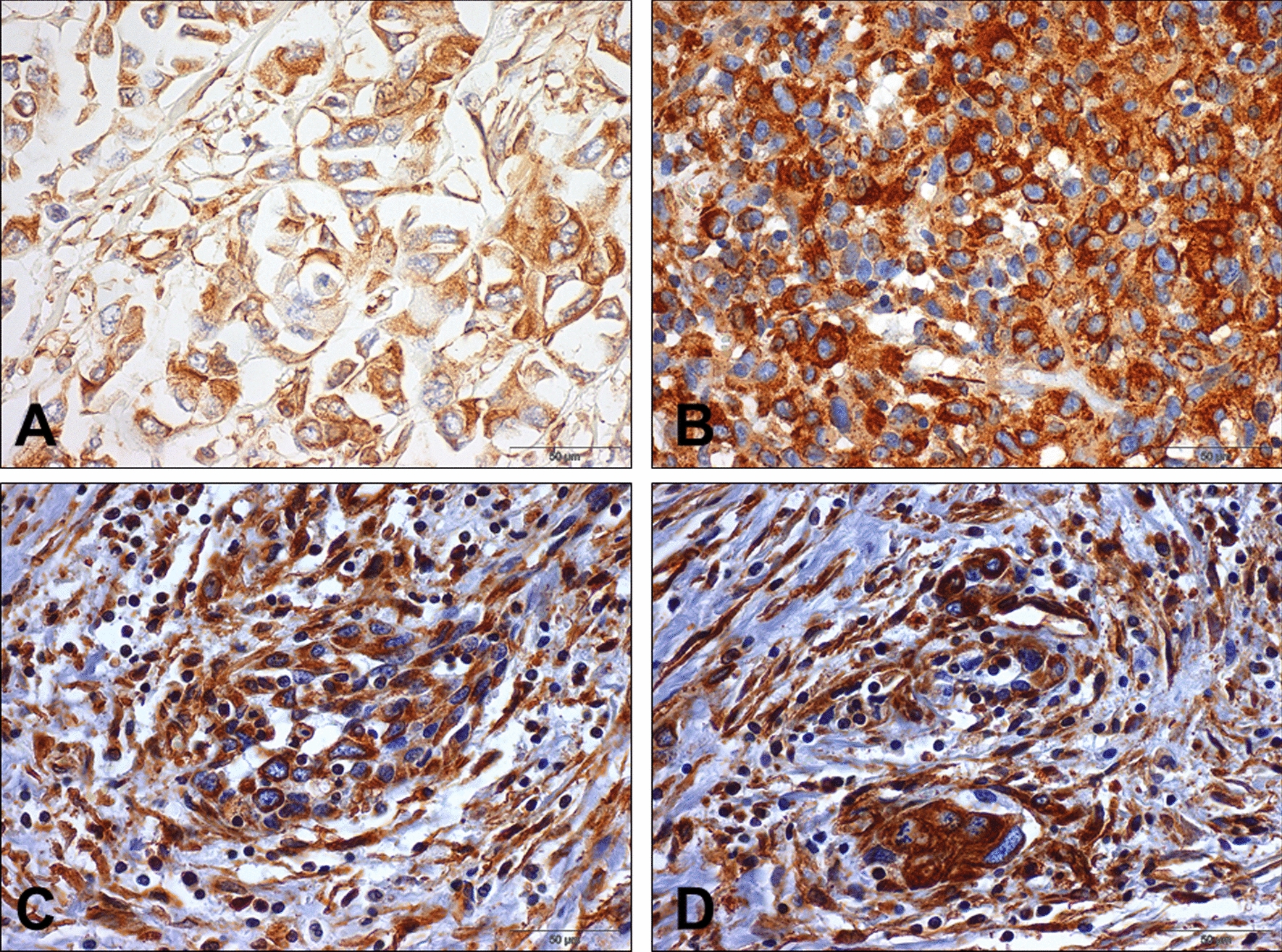
Immunoexpression of vimentin in the bladder cancer cohort. **a**, **b**: immunoexpression of vimentin in primary bladder cancer specimens, one NMIBC (**a**) and one MIBC (**b**); **c** and **d**: immunoexpression of vimentin in bladder cancer metastases

VIM immunoexpression score did not have a significant impact on DSS and DFS for MIBC patients (p = 0.141 and p = 0.512, respectively). It also did not significantly influence DSS of NMIBC patients (p = 0.296). Importantly, however, NMIBC patients with VIM immunoexpression in tumor cells endured significantly worse DFS (p = 0.005, Fig. 4). DFS of NMIBC patients with VIM immunoexpression was significantly poorer (HR = 3.541, 95% confidence interval 1.402–8.943), and this was maintained after adjusting for patients’ age (HR = 3.678, 95% confidence interval 1.435–9.423) and tumor grade (HR = 3.223, 95% confidence interval 1.104–9.408). Illustrative examples of VIM immunoexpression patterns are depicted in Fig. 5.

## Discussion

BlCa remains a clinically challenging disease, owing to heterogeneity in presentation, progression and distinct treatment strategies. On the one hand, NMIBC is the most frequent BlCa phenotype [35], and disease recurrence is very frequent. Substantial research efforts have been put towards uncovering non-invasive, liquid biopsy-based biomarkers for accurately diagnosing and following-up these patients [36, 37]. One major gap in NMIBC relates to patient prognostication and risk stratification after resection, fundamental for establishing the most appropriate follow-up strategy. In this context, tissue biomarkers that predict relapse may be clinically useful, especially if easily and reproductively assessed, by cost-effective methodologies [38]. On the other end of the spectrum, around 20–25% of patients present already with MIBC. This subtype has dismal prognosis and survival has remained overall unchanged in the last couple of decades. Recently, immunotherapy has proved useful in the metastatic setting, with several agents being approved and shown to be effective [39, 40]. However, again, there is a need for better biomarkers predictive of response to specific agents [41, 42], that can be determined in tissue samples upon radical cystectomy and also non-invasively, in liquid biopsy context.

Being such a heterogeneous disease, molecular classification of BlCa was introduced and gained popularity in the past years [10–20]. It is intended to meet these current needs, improving risk stratification of BlCa, and also aiding in identifying specific targets that can be druggable with specific agents. The several analyses concur in the fact that two major types of BlCa are molecularly defined, with important prognostication value: the “luminal” and the “basal” cancers. Such classification is achieved based on genomic and transcriptomic analyses, which point to differential expression of specific markers among tumors: the basal cytokeratins *KRT5/KRT6A* and *KRT14,* as hallmarks of basal BlCa, and the luminal markers *FOXA1* and *GATA3*, as hallmarks of luminal cancer. The value of the classification seems undoubtful; however, and despite multiple confirmations of this, such classification is still not being used in routine clinical practice. There is a lack of works attempting to validate it in the diagnostic setting using immunohistochemistry, with the ones available also finding difficulties in purely classifying the tumors into subtypes or retrieving the same prognostic value [18, 22]. The main aim of our work was to assess the protein expression of these markers and attempt to classify these tumors in a well-defined cohort of BlCa, representative of the diagnostic routine of a tertiary cancer center.

We have witnessed substantial overlapping in protein expression of luminal and basal markers within BlCa specimens, with 40.5% of our cohort showing protein expression of both types of markers. Such overlapping was maintained across both MIBC and NMIBC. We believe that this may be explained by intratumor heterogeneity and specific tumor cell clones within the tumor mass (also acknowledged by Kamoun et al. [10]), which are captured by immunohistochemistry technique, but may go unnoticed in wide transcriptomic analyses. Moreover, we provide data not only on expression patterns in MIBC, but also in NMIBC. The former depicted higher proportion of CK5/6 positive cases (47.2% versus 39.2%), but basal features could be already pinpointed in NMIBC, as well. Although in NMIBC this did not dictate differences in recurrence, it might be due to small size of our cohort; on the same line, the proportion of recurrences in MIBC was higher in cases with CK5/6 expression (32.4% versus 13.2%), again with the lack of significant impact on DFS likely due to small number of cases tested (or simply because of other cohort selection issues, like for Choi et al. [22]). Additionally, the neuroendocrine-like subtype was recently added to the classification [10], and we identified one such case within the four tumors negative for both luminal and basal markers. We hypothesize that the remaining cases might also belong in this category, but they are still changing their program and progressing towards a more pronounced neuroendocrine phenotype. Overall, the classification proposed based on expression of these markers remains informative and has potential to be translated to practice if appropriate definitions and methodologies are set (i.e. accurate definitions of “luminal” and “basal” tumors at the protein level, as determined by immunohistochemistry should be established and validated, in order to maintain the clinical value). Prospective, multicenter studies with systematic evaluation of these markers by the same methodology and reporting system should be instrumental for achieving a consensus. We found significant positive correlations between mRNA expression levels of *GATA3*, *KRT5* and *KRT6A* and the matched immunoexpression scoring for the same markers on the same samples (like in the work of Choi et al. [22]). This also substantiates the applicability of the classification. We hypothesize that the classification could also be extended to upper urothelial tract carcinomas, a work ongoing in our Group, with 15/57 tumors (26.3%) showing CK5/6 immunoexpression (data not shown).

In another setting, the EMT signaling pathway and its players have been implicated in acquisition of a more aggressive cancer phenotype among various tumor models, demonstrated both in vitro, in vivo and validated in clinical studies with human specimens [43, 44]. The role of expression of epithelial markers such as E-cadherin, the phenomenon of cadherin switch and overexpression of mesenchymal markers (like Snail, Twist, ZEB1/2, Slug and VIM) has been shown across tumor models [45–48]. BlCa is no exception, with studies evidencing that mesenchymal features significantly associate with higher propensity for disease recurrence, metastatic spread, tumor progression and worse prognosis, including poorer survival and treatment resistance [31, 33, 49–52]. In this work, we have assessed the role of the intermediate filament *VIM*, characteristic of cells with mesenchymal phenotype, not expressed in most normal epithelia (including urothelium), in predicting prognosis of BlCa patients. In accordance, we have shown that *VIM* mRNA and protein expression levels were significantly higher in MIBC compared to NMIBC, illustrating association with increased stage (Fig. 3a, b). The increase in VIM protein expression within increasingly aggressive samples (Fig. 3c) reflects the influence of EMT in acquisition of a more aggressive phenotype. Finally, translating this to patient outcome, patients with NMIBC disclosing higher VIM expression were shown to have shorter DFS (Fig. 4), even when adjusting (in multivariable analysis) for patient age and grade. Indeed, VIM *de*-*novo* expression or overexpression has been consistently reported in various epithelial cancers, including those of prostate, breast and lung, associating with increased tumor growth, invasion, poor prognosis, and ultimately, with EMT [53–55]. In BlCa, several reports suggest that VIM associates with higher grade and stage [32, 56, 57], and with propensity for recurrence and metastasis; however, vimentin immunohistochemistry is not routinely performed when assessing BlCa specimens. Also, VIM was shown to be expressed in 100% of the cases of sarcomatoid urothelial carcinoma (along with positivity for other mesenchymal markers such as Snail in a high proportion of cases), a particularly aggressive form of the disease, with dismal prognosis [58]. Our work goes further and indicates the clinical potential of VIM as a prognostic marker within luminal vs. basal-like BlCa cases, although larger studies, including both NMIBC and MIBC, are needed to confirm this hypothesis.

Limitations of this work include its retrospective nature, and the relatively low number of samples with complete clinical information available. Also, not all samples in which immunohistochemistry was performed had fresh-frozen material available for performing transcript analyses. Moreover, although immunohistochemistry may be subjected to inter-observer variability, it is a widespread technique, used in routine histopathology, allowing for evaluating morphology simultaneously and perceiving details related to tumor heterogeneity. Importantly, this work also extends the molecular classification to NMIBC, which should be further explored in the future.

## Conclusions

In conclusion, we show that BlCa molecular classification has the potential to be effectively translated to the diagnostic routine, but effort must be made to consistently define the tumor categories acknowledged by transcriptomic studies using routine techniques, with the ultimate goal of maintaining the same clinically meaningful input. On the other hand, expression of EMT markers may be useful for predicting relapse and adjusting therapeutic strategy, like VIM in our work, in which it provided useful prognostic information and dictated survival outcome. Adjunctive markers to the molecular classification merit attention as they might further improve BlCa molecular taxonomy.

## Supplementary information

**Additional file 1: Table** **S1.** Immunohistochemistry methods.

**Additional file 2: Table** **S2.** Primers sequences.

**Additional file 3: Figure** **S1:** Immunoexpression of neuroendocrine markers in a bladder cancer specimen negative for CK5/6, FOXA1 and GATA3. A: CD56; B: Synaptophysin; C: Chromogranin.

## Data Availability

The datasets used and/or analyzed during the current study are available from the corresponding author on reasonable request.

## Abbreviations

BlCa: Bladder cancer
NMIBC: non-muscle invasive bladder cancer
MIBC: Muscle invasive bladder cancer
FGFR3: Fibroblast growth factor receptor 3
EMT: Epithelial-to-mesenchymal transition
VIM: Vimentin
TUR: Transurethral resection
AJCC: American Joint Committee on Cancer
DSS: Disease-specific survival
DFS: Disease-free survival
HR: Hazard ratios
