# Supplementary material for: Practicability of clinical application of bladder cancer molecular classification and additional value of epithelial-to-mesenchymal transition: prognostic value of vimentin expression
Source: J Transl Med. 2020 Aug 5;18:303. doi: 10.1186/s12967-020-02475-w (PMC7405371; doi:10.1186/s12967-020-02475-w)
Supplement: Supplementary file 1 — Additional file 1: Table S1. Immunohistochemistry methods. [file 12967_2020_2475_MOESM1_ESM.docx]

**Supplementary Table 1. Immunohistochemistry methods.**

| Antibody | Clone/Ref | Vendor | Antigenic recovery | Dilution | Detection system | Incubation/Platform | Positive control |
| --- | --- | --- | --- | --- | --- | --- | --- |
| GATA3 | **Polyclonal/HPA029731** | **Sigma** | **Citrate, 20 min** | **1:350** | **Novolink Max Polymer Detection System** | **1h** | **Breast cancer** |
| FOXA1 | **Polyclonal/HPA050505** | **Sigma** | **Citrate, 20 min** | **1:300** | **Novolink Max Polymer Detection System** | **1h** | **Breast cancer** |
| CK5/6 | **Monoclonal (D5/16B4)/355M-18** | **Cell Marque** | **ER2, 20 min** | **1:200** | **Bond Polymer Refine Detection Kit** | **Leica Bond III** | **Squamous cell carcinoma** |
| VIM | **Monoclonal (V9)/NCL-L-VIM-V9** | **Leica** | **ER1, 10min** | **1:100** | **Bond Polymer Refine Detection Kit** | **Leica Bond III** | **Tonsil** |
| Synaptophysin | **Monoclonal (DAK-SYNAP)** | **Dako** | **ER2, 10 min** | **1:100** | **Bond Polymer Refine Detection Kit** | **Leica Bond III** | **Pancreas** |
| Chromogranin | **Monoclonal (DAK-A3)** | **Dako** | **ER1, 10 min** | **1:1000** | **Bond Polymer Refine Detection Kit** | **Leica Bond III** | **Pancreas** |
| CD56 | **Monoclonal (CD564)** | **Leica** | **ER2, 10 min** | **1:100** | **Bond Polymer Refine Detection Kit** | **Leica Bond III** | **Pancreas** |
