# Supplementary material for: Practicability of clinical application of bladder cancer molecular classification and additional value of epithelial-to-mesenchymal transition: prognostic value of vimentin expression
Source: J Transl Med. 2020 Aug 5;18:303. doi: 10.1186/s12967-020-02475-w (PMC7405371; doi:10.1186/s12967-020-02475-w)
Supplement: Supplementary file 2 — Additional file 2: Table S2. Primers sequences. [file 12967_2020_2475_MOESM2_ESM.docx]

**Supplementary Table 2. Primers sequences.**

| Gene | Forward (5’-3’) | Reverse (5’-3’) |
| --- | --- | --- |
| *GATA3* | CAGACCACCACAACCACACTCT | GGATGCCTCCTTCTTCATAGTCA |
| *FOXA1* | GGGTGGCTCCAGGATGTTAGG | GGGTCATGTTGCCGCTCGTAG |
| *KRT5* | ATCGCCACTTACCGCAAGCTGCTGGAGGG | AAACACTGCTTGTGACAACAGAG |
| *KRT6A* | AGAGAATGAATTTGTGACTCTGAAGAAG | TACAAGGCTCTCAGGAAGTTGATCT |
| *GUSB* | CACTGAAGAGTACCAGAAAAGTC | TCTCTGCCGAGTGAAGATCC |
