# Supplementary figures and images for: Practicability of clinical application of bladder cancer molecular classification and additional value of epithelial-to-mesenchymal transition: prognostic value of vimentin expression
Source: J Transl Med. 2020 Aug 5;18:303. doi: 10.1186/s12967-020-02475-w (PMC7405371; doi:10.1186/s12967-020-02475-w)

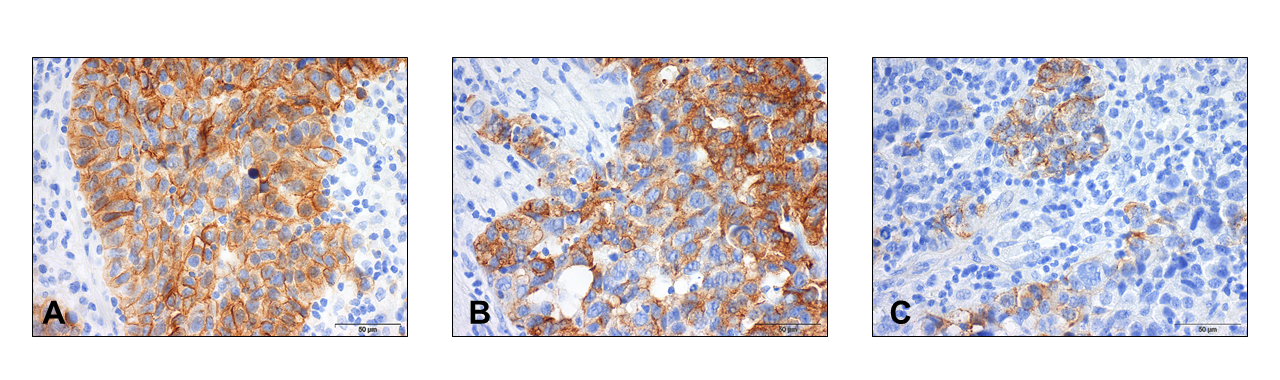

Supplement: Supplementary file 3 — Additional file 3: Figure S1: Immunoexpression of neuroendocrine markers in a bladder cancer specimen negative for CK5/6, FOXA1 and GATA3. A: CD56; B: Synaptophysin; C: Chromogranin. [file 12967_2020_2475_MOESM3_ESM.tif]
